# Population Pharmacokinetic Model-Based Evaluation of Intact Oxaliplatin in Rats with Acute Kidney Injury

**DOI:** 10.3390/cancers13246382

**Published:** 2021-12-20

**Authors:** Shinji Kobuchi, Miyu Kai, Yukako Ito

**Affiliations:** Department of Pharmacokinetics, Kyoto Pharmaceutical University, Kyoto 607-8414, Japan; kobuchi@mb.kyoto-phu.ac.jp (S.K.); miiyu717@gmail.com (M.K.)

**Keywords:** renal dysfunction, pharmacokinetic modeling and simulation, platinum compounds, cancer chemotherapy

## Abstract

**Simple Summary:**

Acute kidney injury (AKI) complicates the dose setting of oxaliplatin (L-OHP), making it difficult to continue treatment cycles and retain antitumor efficacies with minimum L-OHP-related toxicities. Our study aimed to assess the impact of AKI on the pharmacokinetics of intact L-OHP and simulate the relationship between the degree of renal function and intact L-OHP exposures using a population pharmacokinetic model. Mild and severe renal dysfunction model rats were used to determine plasma and urine intact L-OHP concentration–time profiles after L-OHP administration. No significant differences in intact L-OHP levels between rats with normal renal function and those with renal dysfunction were observed, whereas renal excretion of intact L-OHP was correlated with renal function. Results of population PK model simulation suggested that dose reduction is dispensable for patients with mild to moderate AKI. The population PK modeling and simulation approach can contribute to developing an appropriate dose regimen of L-OHP for AKI patients.

**Abstract:**

Acute kidney injury (AKI) complicates the dosing strategies of oxaliplatin (L-OHP) and the requirement for L-OHP dose reduction in patients with renal failure remains controversial. The objective of this study is to assess the impact of AKI on the pharmacokinetics (PK) of intact L-OHP and simulate the relationship between the degree of renal function and intact L-OHP exposures using a population PK model. Intact L-OHP concentrations in plasma and urine after L-OHP administration were measured in mild and severe AKI models established in rats through renal ischemia-reperfusion. Population PK modeling and simulation were performed. There were no differences among rats in the area under the plasma concentration–time curve of intact L-OHP after intravenous L-OHP administrations. Nevertheless, the amount of L-OHP excretion after administration of 8 mg/kg L-OHP in mild and severe renal dysfunction rats was 63.5% and 37.7%, respectively, and strong correlations were observed between biochemical renal function markers and clearance of intact L-OHP. The population PK model simulated well the observed levels of intact L-OHP in AKI model rats. The population PK model-based simulation suggests that dose reduction is unnecessary for patients with mild to moderate AKI.

## 1. Introduction

Acute kidney injury (AKI) is a common and critical complication in the treatments of cancer [1]. According to the guidelines, AKI is classified, and its management is performed based on plasma creatinine (Cr) levels, glomerular filtration rate, and urine volume [2]. A series of pathological processes involved in AKI results in decreased glomerular filtration and renal excretion [3,4], leading to a decrease in renal clearance of drugs and toxins [5]. In addition, AKI also affects the disposition and hepatic clearance of drugs and toxins [6,7,8], complicating our understanding of drug pharmacokinetics (PK). For cancer patients with AKI, the risk of toxicity from chemotherapeutic agents is increased, leading to the discontinuation of cancer chemotherapy or the change to alternative treatments. AKI requires a dose setting for each patient and dose reduction in chemotherapy. Excessive dose reduction based on the physician’s experience may induce attenuation of antitumor effects. Therefore, to continue the treatment cycle and retain the antitumor efficacy, the development of a rational dosing strategy based on renal function is needed but remains a challenge.

Oxaliplatin (L-OHP) is a third-generation platinum (Pt)-based anticancer agent that is widely prescribed for patients with colorectal, pancreatic, or stomach cancer [9,10,11]. Regardless of the desirable antitumor efficacy of L-OHP, peripheral neuropathy is a major adverse effect and dose-limiting toxicity [12,13]. Unlike side effects of cisplatin which is also a Pt-based anticancer agent, renal failure is uncommon because of the low accumulation of L-OHP in the renal tubular epithelial cells [14,15,16]. Thus, extensive hydration before the start of chemotherapy is not required [17] and dose modifications are not common in patients with renal disorders (creatinine clearance (C_Cr_) > 20 mL/min) since L-OHP is excreted via the kidneys [18,19]. With growing the number of prescriptions of L-OHP due to its lower risk of AKI than with other Pt-based agents, the reports of L-OHP-related AKI cases are increasing [20]; however, the rational dose modifications for patients with AKI within the sequential chemotherapeutic cycles has not been established.

The requirement for L-OHP dose modification in patients with renal dysfunction including AKI remains controversial. A recent retrospective study reported that renal failure (C_Cr_ < 60 mL/min) is a risk factor for L-OHP-related toxicity and appropriate intervention is needed in patients with renal disorders [21]. In another previous study, the standard dose of L-OHP was applied in S-1 plus L-OHP combination chemotherapy for advanced gastric cancer patients both with (30 ≤ C_Cr_ < 60 mL/min) and without renal failure (C_Cr_ ≥ 60 mL/min), and its efficacy and safety were reported [22]. However, these studies have limitations, being retrospective and performed in a single institute with a small sample size and no pharmacokinetic (PK) assessments [21,22]. Although the effects of chronic kidney disease (CKD) on PK of anticancer agents in patients have been extensively investigated, the impact of AKI on PK of L-OHP remains still unknown. To find whether there is a necessity for L-OHP dose modification in patients with AKI and to construct the dosage determination criteria, PK of L-OHP in AKI should be evaluated.

L-OHP is rapidly converted into several biotransformation products after administration [23] that then irreversibly bind to macromolecules in vivo [24,25], leading to inactivation [26]. To assess the PK of L-OHP, total platinum (Pt) concentrations are generally measured due to difficulties in determining the level of each product with high sensitivity in clinical research; however, these assessments may lead to wrong interpretations. To evaluate the effects of AKI on the PK of L-OHP, intact L-OHP should be distinguished from biotransformation products [27,28]. Therefore, the current study evaluated the impact of the degree of AKI on intact L-OHP exposure in renal ischemia-reperfusion model rats and simulated exposure using population PK model analysis.

## 2. Materials and Methods

### 2.1. Chemicals and Animals

L-OHP was obtained from Wako Pure Chemical Industries, Ltd. (Osaka, Japan). Elplat^®^ was supplied by Yakult Honsha Co., Ltd. (Tokyo, Japan). All reagents were at least of analytical grade. Ten-week-old male Wistar rats were purchased from Nippon SLC Co., Ltd., Hamamatsu, Japan. The rats were housed in a temperature-controlled room under a 12 h light/dark cycle and allowed free access to standard rat chow and water. All animal experimental procedures were approved by the institutional review board and conducted following the Kyoto Pharmaceutical University Guidelines for Animal Experimentation.

### 2.2. Renal Failure Models

A previously established animal model of renal ischemia-reperfusion injury was used [8]. Briefly, a midline abdominal incision was performed to expose the kidneys under intraperitoneal anesthesia using a mixture of 0.375 mg/kg medetomidine, 2.0 mg/kg midazolam, and 2.5 mg/kg butorphanol. The renal artery and vein were isolated and blood flow was occluded by non-traumatic microvascular clamps around both renal arteries. The degree of renal failure was classified into mild and severe renal dysfunction groups by ischemic time. After 30 min (mild renal dysfunction group) or 60 min (severe renal dysfunction group), the artery clamps were removed, and reperfusion was allowed for 24 h. The success of ischemia and reperfusion was confirmed by color changes of the renal surface. The incision was closed with a skin stapler and the renal failure model rats were used for experiments after 24 h reperfusion. Control rats were prepared using the same procedure except for the clamp. To measure the biochemical parameters at Kyoto Biken Laboratories Inc. (Kyoto, Japan), blood samples were collected from the left jugular vein before the surgical procedure and 24 h post-reperfusion. The value of creatinine (Cr) in plasma and urine sample was determined using LabAssay^TM^ Creatinine (Wako Pure Chemical Industries, Ltd., Osaka, Japan). C_Cr_ was calculated by dividing the amount of creatinine excreted into urine samples by its plasma concentration.

### 2.3. PK Study of Intact L-OHP

All rats (*n* = 30) were divided into three groups according to the degree of renal failure as control, mild, and severe renal failure. L-OHP (Elplat^®^, 5 mg/mL) was administered intravenously to the jugular vein at a dosage of 3 or 8 mg/kg (*n* = 5 in each dose group), using the dosage determined based on clinical doses and previous animal studies [29]. Blood samples were collected from the jugular vein at 3, 5, 10, 20, 30, 45 min, 1, 1.5, and 2 h after dosing. Urine samples were collected via bladder catheterization at 0–0.5, 0.5–1, 1–1.5, 1.5–2, 2–3, and 3–4 h after 8 mg/kg L-OHP administration. Due to the limit of quantitation of L-OHP, drug levels in urine samples were determined only in the high-dose group for rats. To precipitate the plasma protein, the obtained blood sample was immediately centrifugated at 14,000× *g* for 3 min and acetonitrile was added to obtained plasma sample. Collected urine samples were also precipitated by acetonitrile. Intact L-OHP concentration in plasma and urine was measured by liquid chromatography-tandem mass spectrometry (LC-MS/MS) using the method reported by Ito et al. [30]. The details of the LC-MS/MS assay procedure have been described in our previous reports [31].

### 2.4. Population PK Model Analysis

Plasma L-OHP concentration data were analyzed using a non-linear mixed-effect modeling software, Phoenix^®^ NLME^TM^ Version 8.2 (Certara USA, Inc., Princeton, NJ, USA). Before the population pharmacokinetic analysis, non-compartmental pharmacokinetic analysis (NCA) was performed using the plasma L-OHP concentration data with the NCA program of Phoenix WinNonlin^®^ software (version 8.2, Certara USA, Inc., Princeton, NJ, USA). The area under the plasma concentration–time curve from the time of dosing to infinity (*AUC*_0–∞_) was determined by the linear trapezoidal rule. The half-life (*t*_1/2_) was calculated by the terminal slope (*k*_e_) determined by the linear regression from the terminal phase of the plasma concentration–time curve. Total clearance (*CL*_tot_) was calculated by dose/*AUC*_0–∞_, and distribution volume (*Vd*) was determined by *CL*_tot_/*k*_e_. 

In population analysis, to obtain the population parameters and their variabilities, the first-order conditional estimation with extended least squares (FOCE-ELS) method was applied using Phoenix^®^ NLME^TM^ Version 8.2 software (Certara USA, Inc., Princeton, NJ, USA). The 2-compartment model with a linear elimination was selected as a PK model based on the experimental data. The inter-individual and residual variability of models were determined according to −2 × log-likelihood (−2LL), goodness-of-fit (GOF) plots, and the coefficient of variation (CV) of parameter estimates. The individual pharmacokinetic parameters were assumed by the exponential error model. A proportional error model was used to determine the residual variability in the observed L-OHP concentrations. 

For validation of the final model, graphical and statistical methods were used. The GOF plots are graphical summaries that describe observed concentration (OBS) vs. population predicted concentration (PRED) and OBS vs. individual population predicted concentration (IPRED). GOF plots include conditional weighted residuals (CWRES) vs. PRED and conditional weighted residuals (CWRES) vs. time after dose (TAD). In addition, a prediction-corrected visual predictive check (pcVPC) (*n* = 1000) and a nonparametric bootstrap procedure (*n* = 1000) were also conducted. The pcVPC can remove the independent variables (dose and other covariates) and evaluate the performance of the final PK model [32]. The current observed data used for model analysis were obtained from the PK study with two doses (3 or 8 mg/kg L-OHP); then, the predictive performance of the model was assessed using pcVPC. To check the model stability and predictability, the original data set was checked by overlaying with the 5th and 95th percentiles of the model-based simulation data. The median and 5th and 95th percentiles of the observed data were compared with the corresponding percentiles of the simulated data. In the bootstrap procedure, the median and 95th percentile confidence intervals (95th CI) of estimates obtained from the bootstrap data sets were compared with those obtained from the original data set.

### 2.5. Population PK Model-Based Simulation

To simulate the impact of renal failure on L-OHP disposition, simulated plasma L-OHP concentration curves were plotted relative to different plasma Cr levels. As the best correlation between Cr level and post hoc *CL* of L-OHP, plasma L-OHP concentrations were simulated (*n* = 1000) using the Monte Carlo method and the final population pharmacokinetic model parameters including the *CL* determined by the regression equations of Cr level and post hoc *CL*. According to the previously reported plasma Cr levels in rats with renal failure model [33], the model-based simulation was performed with plasma Cr levels ranging from 0.3 to 2.5 mg/dL.

## 3. Results

### 3.1. Biochemical Parameters in Renal Failure Model Rats

Biochemical parameters of normal, mild, and severe renal failure model rats are shown in Table 1. The amounts of albumin in plasma of mild and severe renal failure model rats were significantly lower than those in normal rats. AST and ALT levels were significantly increased in severe renal failure model rats. As renal functions, BUN levels in severe renal failure model rats were higher than that in normal rats and Cr levels were increased dependently with ischemic time. C_Cr_ values also decreased with increasing ischemic time.

### 3.2. NCA Analysis and Urinary Excretion of Intact L-OHP in Renal Failure Model Rats

**Figure 1:**
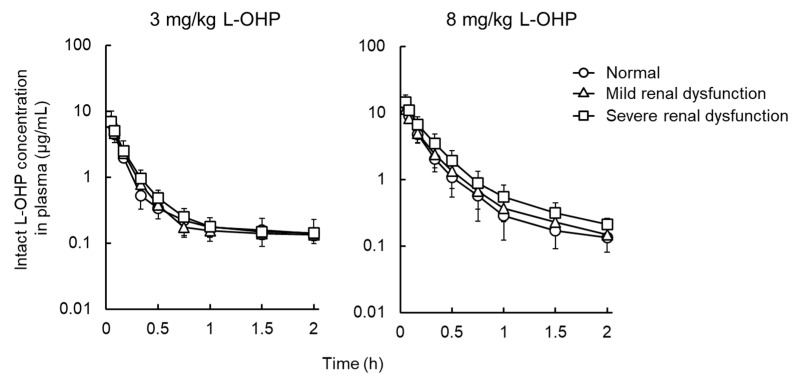
Mean plasma concentration profiles of oxaliplatin (L-OHP) after intravenous administration of 3 or 8 mg/kg of L-OHP. Each symbol represents normal (○), mild (△), or severe renal dysfunction (□) groups. Each point shows the mean ± S.D. (*n* = 4–5).

**Figure 2:**
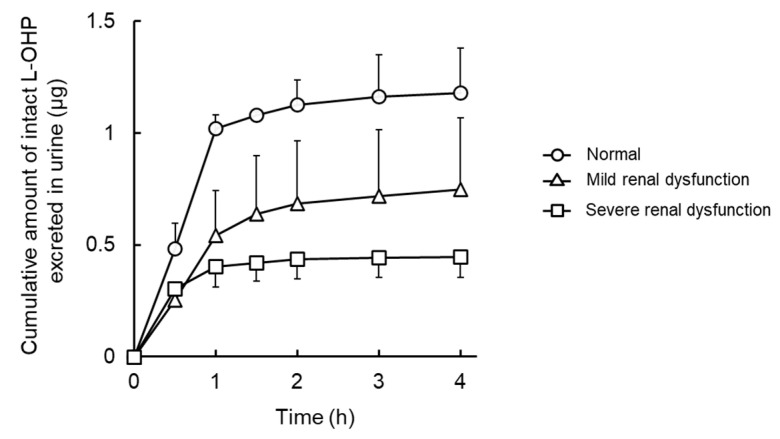
Mean cumulative urinary excreted amounts of oxaliplatin (L-OHP) versus time after intravenous administration of 8 mg/kg of L-OHP in normal, mild, and severe renal dysfunction model rats. Each symbol represents normal (○), mild (△), or severe renal dysfunction (□) groups. Each point shows the mean ± S.D. (*n* = 3–4).

**Figure 3:**
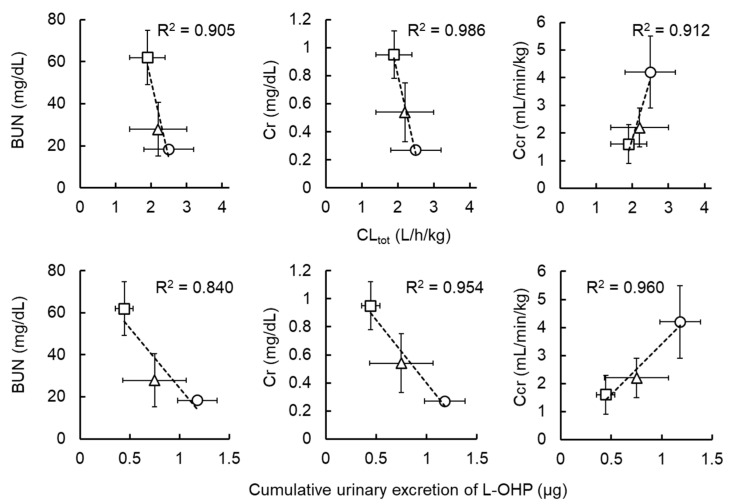
Correlation between biochemical parameters (blood urea nitrogen (BUN), creatinine (Cr), creatinine clearance (C_Cr_)), and pharmacokinetic parameters of oxaliplatin (L-OHP) (total clearance (CL_tot_) and cumulative urinary excretion of L-OHP for 4 h). Each symbol represents normal (○), mild (△), or severe renal dysfunction (□) groups. Each point shows the mean ± S.D. (*n* = 3–5).

Figure 1 shows the plasma concentration–time curve of L-OHP after intravenous administration of L-OHP to normal, mild, and severe renal dysfunction model rats. Figure 2 presents cumulative excretion of L-OHP after a bolus injection of 8 mg/kg L-OHP. PK parameters determined by NCA analysis are listed in Table 2. There were differences in *t*_1/2_ values between the 3 mg/kg and 8 mg/kg L-OHP groups, possibly due to the short sampling time in the 8 mg/kg L-OHP group. In the mild and severe renal dysfunction groups, slightly low *CL*_tot_ was observed in a dysfunction grade-dependent manner, whereas results of all PK parameters in NCA analysis presented no significant alterations between normal and renal dysfunction models. The cumulative amount of intact L-OHP excreted in urine up to 4 h after administration of 8 mg/kg L-OHP (i.e., 2400 μg for 300 g weight of rats) was 1.2 ± 0.2 μg and the urinary excretion rate of intact L-OHP was estimated to be <0.1%. The amount of L-OHP excretion after administration of 8 mg/kg L-OHP in mild and severe renal dysfunction was 63.5% and 37.7% of rats with normal renal function. Figure 3 shows the correlation between biochemical parameters and PK parameters after administrations of 8 mg/kg L-OHP. There were strong correlations between biochemical markers representing renal function (BUN, Cr, and C_Cr_) and *CL*_tot_ after administrations of 8 mg/kg L-OHP. These markers also correlated with cumulative urinary excretion of L-OHP. Although C_Cr_ was the most strongly correlated marker for cumulative urinary excretion of L-OHP, Cr was the most reflective marker for *CL*_tot_.

### 3.3. Population PK Analysis of Intact L-OHP in Normal and Renal Failure Model Rats

A two-compartment model with a linear elimination described well the PK of intact L-OHP in normal, mild, and severe renal dysfunction models. Final PK model diagnostic plots are presented in Figure S1. The population PK parameter estimates and results of the bootstrap validation are shown in Table 3. The modeling-based fitting enables simulation of the PK profiles and improves reliability by comparing the parameters of NCA analysis to the estimated PK model parameters. In this model, distribution volume in the central (*V*_1_) and peripheral compartments (*V*_2_) were estimated as 0.44 and 2.26 L/kg (*Vd*: 2.70 L/kg), respectively, that are comparable with *Vd* values (2.2–5.8 L/kg) determined by NCA analysis. The clearance from the central compartment (*CL*: 1.76 L/h/kg) and inter-compartmental clearance (*CL*_2_: 1.0 L/h/kg) (*CL*_tot_: 2.76 L/h/kg) were similar to the *CL*_tot_ (1.3–2.5 L/h/kg) value calculated by NCA analysis. The coefficient of variation (CV%) of all PK parameter estimates ranged from 6.9% to 26.0%, indicating that model precision was acceptable. The inter-individual variability parameters were *V* (37.5%), *CL* (30.5%), and *CL*2 (31.5%). Based on the −2LL value and the GOT plots, the proportional error was best for the plasma concentration of L-OHP. 

**Figure 4:**
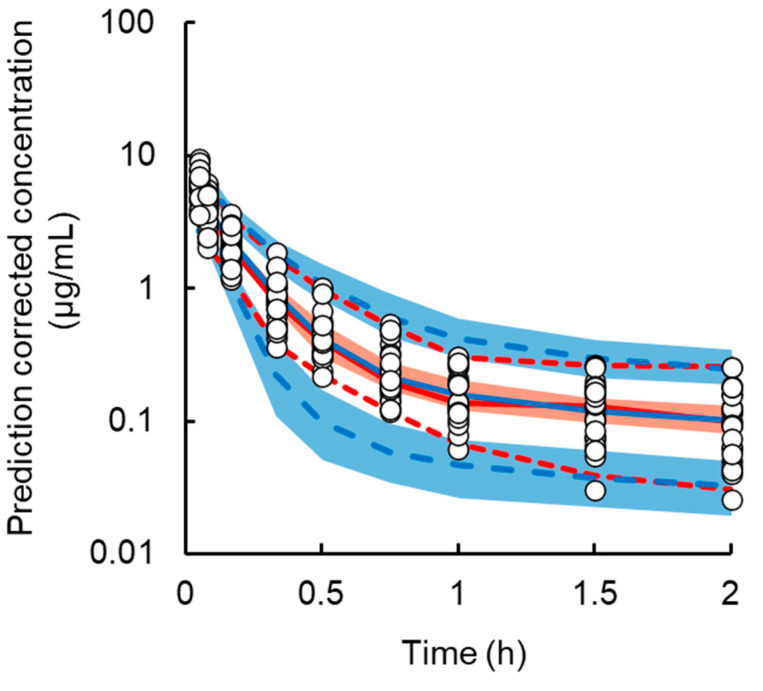
Prediction-corrected visual predictive check (pcVPC) plot for the final pharmacokinetic model of oxaliplatin (L-OHP) in normal, mild, and severe renal dysfunction model rats. The open circles represent individual observations. The red solid line represents the median and dashed lines represent the 5th and 95th percentiles of the observations, respectively. The blue solid line represents the median and dashed lines represent the 5th and 95th percentiles of the predictions, respectively. The shaded red area represents the 95% confidence interval of the median and the blue areas represent the 2.5th and 97.5th percentiles of the predictions, respectively.

The median value of each model parameter estimate from the bootstrap procedure was similar to that from the original data set, and the 95th CI of the estimated value from bootstrap was narrow. The results of pcVPC of the PK model are presented in Figure 4 and show that the observed values were largely overlayed within the median, 5th, and 95th percentiles of the simulation data. These validation results indicate that this population PK model was robust and sufficient for simulating plasma L-OHP concentrations.

### 3.4. Simulations to Assess the Impact of Renal Failure on Plasma Concentration of L-OHP

**Figure 5:**
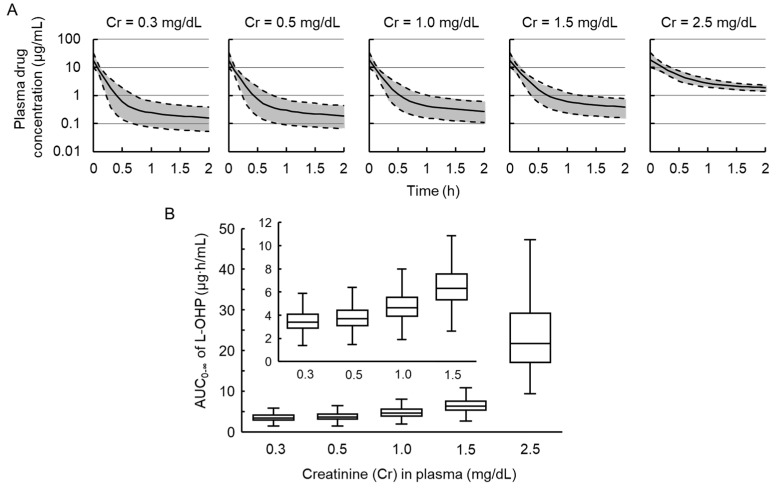
Simulated plasma concentration profiles and exposures of oxaliplatin (L-OHP) in groups with different plasma creatinine (Cr) levels (*n* = 1000). (**A**) Plasma L-OHP concentration vs. time curves after intravenous administration of 8 mg/kg of L-OHP in rats. The solid black line represents the simulated median plasma concentration, and the semitransparent gray field represents the 5th and 95th percentiles of the 1000 data sets simulated from the final population pharmacokinetic model; (**B**) boxplot with the median and interquartile range of the area under the plasma concentration–time curve of L-OHP from time of dosing to infinity (*AUC*_0–∞_) after intravenous administration of 8 mg/kg of L-OHP in rats according to plasma Cr levels.

To estimate the impact of renal failure on plasma L-OHP levels, the median, 5th, and 95th percentiles of plasma L-OHP concentrations following intravenous administration of 8 mg/kg L-OHP were simulated by the population PK model and Monte Carlo simulation method and are shown in Figure 5. The median (5th–95th percentiles) of *AUC*_0–__∞_ in Cr = 0.3, 0.5, and 1.0 mg/dL was 3.4 (2.2–5.3), 3.7 (2.4–5.7), and 4.6 (3.0–7.2) μg∙h/mL, respectively, comparable with the *AUC*_0–__∞_ values from observed data in normal (3.4 ± 0.9 μg∙h/mL), mild (3.3 ± 1.0 μg∙h/mL), and severe renal failure rats (4.4 ± 1.1 μg∙h/mL). The estimated medians of *AUC*_0–__∞_ in 1.5 (median: 6.3, 5th–95th percentiles: 4.1–9.8 μg∙h/mL) and 2.5 mg/dL (median: 21.7, 5th–95th percentiles: 13.8–42.5 μg∙h/mL) of plasma Cr level were approximately two and seven times higher, respectively, than that in normal renal function group (0.3 mg/dL of Cr).

## 4. Discussion

The effects of renal dysfunction, including CKD and AKI, on the PK of anticancer agents have been widely investigated. L-OHP exhibits a lower risk for nephrotoxicity than cisplatin and dose modification is uncommon in L-OHP-based chemotherapy. However, results of recent clinical studies suggest that renal failure is a risk factor for L-OHP-related toxicity [21], raising the necessity for dose reduction in patients with renal dysfunction to maintain the therapeutic effects of L-OHP and minimize the toxicities. To develop an appropriate dose modification criterion, PK of L-OHP in renal dysfunction should be evaluated. The current study focused on the impact of AKI on the PK of intact L-OHP. Population PK analysis was performed in AKI model rats and used to simulate the exposure of intact L-OHP according to the grade of renal dysfunction using plasma Cr levels. AKI was induced in rats to establish mild and severe kidney dysfunction by inflicting renal ischemic injury for 30 min or 60 min, respectively. The decrease in the C_Cr_ levels in a time-dependent manner was confirmed, indicating the successful establishment of mild or severe renal dysfunction. Moreover, biochemical parameters of Alb, AST, and ALT showed that hepatic dysfunction was also induced with AKI, consistent with previous reports [8,34]. 

The results of this PK study indicate that AKI decreased the renal clearance of intact L-OHP. However, there was only a slight elevation of plasma intact L-OHP levels in severe renal dysfunction. This comparable exposure to intact L-OHP can be related to the extremely low rate of urinary excretion of intact L-OHP. In a previous clinical study, decreased plasma ultrafiltrate Pt clearance and enhanced systemic exposure of Pt were observed in cancer patients treated with single-agent L-OHP, whereas a corresponding increase in L-OHP-related toxicities was not observed [19]. These observations and our results suggest that monitoring the level of intact L-OHP might help in predicting the degree of toxicodynamics of L-OHP in patients with renal dysfunction. 

A population model composed of two compartments can describe the individual and population predictions of the plasma concentration of intact L-OHP in normal and AKI model rats. Previously reported pharmacokinetic studies of L-OHP developed two- or three-compartment models using Pt concentrations in the plasma of rats [35,36,37] or patients [38,39]. Simulation based on the current population PK model with plasma Cr levels revealed that intact L-OHP exposures vary according to the degree of renal function observed in AKI model rats. However, in severe renal dysfunction, i.e., when the Cr levels in plasma were 2.5 mg/dL, the intact L-OHP might be eliminated from plasma slowly, leading to high drug exposures. The current clinical dose strategy of L-OHP for patients with CKD involving full doses at 130 mg/m^2^ are tolerated well in patients with mild-to-moderate renal impairment (C_Cr_ >20 mL/min) and do not result in increased drug-related toxicity [18]. Our modeling and simulation data using intact L-OHP concentrations suggest that dose setting of L-OHP for patients with AKI could be determined based on the dosing strategy for patients with CKD; the dose reductions of L-OHP were found to be unnecessary in patients with mild to moderate AKI who were administered a single agent. However, to elucidate this recommendation, further clinical studies are needed in patients with AKI.

Watanabe et al. recently reported that, in patients with metastatic colorectal cancer who received the regular initial dose of L-OHP in the first cycle, there were no significant differences in the overall survival and incidence rates of adverse events including peripheral neuropathy (grade ≥ 2), neutropenia (grade ≥ 3), and thrombocytopenia (grade ≥ 2) between patients with normal (C_Cr_ ≥ 60 mL/min) and impaired renal function (C_Cr_ < 60 mL/min) [40]. These authors recommended that an initial dose of L-OHP should not be reduced based on the renal function; however, they mentioned the lack of pharmacokinetic data as a limitation of the study. Although the drug efficacy and toxicity were not investigated, our modeling and simulation approach revealed that intact L-OHP exposures in AKI model rats were comparable with that in normal rats. These pharmacokinetic data may support clinical outcomes in patients with renal impairments. 

In the present study, population PK model analysis utilizing intact L-OHP concentrations in plasma of AKI model rats revealed that the exposure to intact L-OHP in mild to moderate AKI is comparable with that in normal renal function. These results could help in establishing the dosing strategy of L-OHP in patients with AKI. The current analysis also sheds light on ambiguities of previously obtained data and may be useful for explaining why there was no relationship between pharmacodynamics/toxicodynamics of L-OHP and renal function in patients with renal dysfunction, unlike with Pt therapy. However, there are several limitations in the current study. Firstly, Pt disposition including total and ultrafiltrate plasma sample was not evaluated in AKI model rats. Simultaneous measurements of both intact L-OHP and Pt are difficult due to limited blood sampling volume in AKI model rats. Secondly, conversion of intact L-OHP to biotransformation products in the urine sample within the experiments may lead to underestimating the urine excretion rate of the drug. Third, we evaluated PK characteristics of intact L-OHP after only the single-agent administration, but not after combination chemotherapy with other anticancer or antiemetic agents. Therefore, further assessment of PK and clinical outcomes including anticancer effects and toxicities at combination chemotherapy are required. Finally, the current results obtained from animals could not be applied to AKI patients directly. To develop the dosing strategy of L-OHP for patients with AKI, further population PK analysis based on the current results must be conducted in clinical studies.

## 5. Conclusions

We investigated the PK of intact L-OHP in mild and severe AKI model rats and developed a population PK model for simulating the quantitative relationship between renal function and intact L-OHP exposures. The novelty of the current study is that intact L-OHP exposures in mild and severe AKI model rats were comparable with that in normal rats, whereas the renal excretion of intact L-OHP was significantly reduced in rats with renal failure. A population PK model that can describe features of plasma intact L-OHP concentration profiles in AKI model rats was successfully developed. Simulation results from the current population PK model suggest that dose reduction of L-OHP would not be required in patients with mild to moderate AKI. It is likely that such an approach involving population PK modeling and simulation would also be applicable to the analysis of clinical data, which can contribute to the development of dosing strategies of L-OHP for AKI patients and to understanding the relationship between PK and pharmacodynamics/toxicodynamics of L-OHP in patients with renal failure. However, to establish an appropriate L-OHP dosing regimen for patients with AKI, further clinical studies investigating both PK and clinical outcomes are needed.

## Figures and Tables

**Table 1:** Biochemical parameters of normal, mild, and severe renal failure model rats.

Biochemical Parameters	Normal	Mild Renal Failure	Severe Renal Failure
TP (g/dL)	6.6 ± 0.4	5.6 ± 0.4 *	6.1 ± 0.4
Alb (g/dL)	4.5 ± 0.2	3.5 ± 0.4 *	3.7 ± 0.2 *
AST (IU/L)	91.7 ± 31.3	473.8 ± 392.7	353.8 ± 150.0 *
ALT (IU/L)	49.8 ± 21.0	88.3 ± 49.5 *	99.5 ± 38.4 *
BUN (mg/dL)	18.3 ± 1.4	27.9 ± 12.7	62.0 ± 12.8 *
Cr (mg/dL)	0.27 ± 0.02	0.54 ± 0.21 *	0.95 ± 0.17 *
C_Cr_ (mL/min/kg)	4.2 ± 1.3	2.2 ± 0.7 *	1.6 ± 0.7 *

Each value represents the mean ± S.D. of four to six rats. TP, total protein; Alb, albumin; AST, aspartate aminotransferase; ALT, alanine aminotransferase; BUN, blood urea nitrogen; Cr, creatinine in plasma; C_Cr_, creatinine clearance. * *p* < 0.05 statistically significant difference vs. the normal rats.

**Table 2:** Non-compartmental pharmacokinetic parameters of oxaliplatin (L-OHP) in normal, mild, and severe renal failure model rats.

Pharmacokinetic Parameters	Normal	Mild Renal Failure	Severe Renal Failure
3 mg/kg L-OHP			
*t*_1/2_ (h)	2.6 ± 1.1	2.8 ± 0.8	2.3 ± 1.6
*AUC*_0–∞_ (μg∙h/mL)	2.0 ± 0.3	2.2 ± 0.2	2.4 ± 0.7
*CL*_tot_ (L/h/kg)	1.6 ± 0.3	1.4 ± 0.1	1.3 ± 0.4
*Vd* (L/kg)	5.8 ± 2.4	5.7 ± 1.9	3.8 ± 1.7
8 mg/kg L-OHP			
*t*_1/2_ (h)	0.7 ± 0.2	0.7 ± 0.2	0.9 ± 0.2
*AUC*_0–∞_ (μg∙h/mL)	3.4 ± 0.9	3.3 ± 1.0	4.4 ± 1.1
*CL*_tot_ (L/h/kg)	2.5 ± 0.7	2.2 ± 0.8	1.9 ± 0.5
*Vd* (L/kg)	2.4 ± 0.9	2.9 ± 1.9	2.2 ± 1.0

Each value represents the mean ± S.D. of four to five rats. *t*_1/2_, elimination half-life; *AUC*_0__–__∞_, area under the plasma concentration–time curve from time of dosing to infinity; *CL*_tot_, total plasma clearance; *Vd*, distribution volume; *MRT*, mean residence time.

**Table 3:** Population pharmacokinetic parameters of oxaliplatin (L-OHP) in normal, mild, and severe renal failure model rats.

Parameters	Unit		Final Model		Bootstrap (*n* = 1000)	
			Estimate	CV%	Median	2.5th–97.5th
Fixed effect parameters (θ)						
*V*		L/kg	0.44	7.4	0.44	0.39–0.49
*V*₂		L/kg	2.26	19.1	2.28	1.62–3.16
*CL*		L/h/kg	1.76	8.8	1.76	1.50–2.03
*CL*₂		L/h/kg	1.0	10.0	1.0	0.84–1.10
Inter-individual variability (ω)						
*V*		%	37.5	19.3	37.1	29.6–43.9
*CL*		%	30.5	26.0	29.8	21.6–37.5
*CL*₂		%	31.5	18.7	31.0	24.8–36.6
Residual variability (σ)						
*C*		%	14.9	6.9	14.9	13.1–17.0

*V*, distribution volume of the central compartment; *V*_2_, distribution volume of the peripheral compartment; *CL*, clearance from the central compartment; *CL*_2_, inter-compartmental clearance; *C*, plasma drug concentration.

## Data Availability

The data presented in this study are available on request from the corresponding author.

## Supplementary Materials

The following are available online at https://www.mdpi.com/article/10.3390/cancers13246382/s1, Figure S1: Final pharmacokinetics model diagnostic plots of the observed versus predicted concentrations and the conditional residuals versus predicted concentrations.
