# Supplementary material for: Population Pharmacokinetic Model-Based Evaluation of Intact Oxaliplatin in Rats with Acute Kidney Injury
Source: Cancers (Basel). 2021 Dec 20;13(24):6382. doi: 10.3390/cancers13246382 (PMC8699120; doi:10.3390/cancers13246382)
Supplement: Supplementary file 1 [file cancers-13-06382-s001.zip › cancers-1510881-supplementary.pdf]

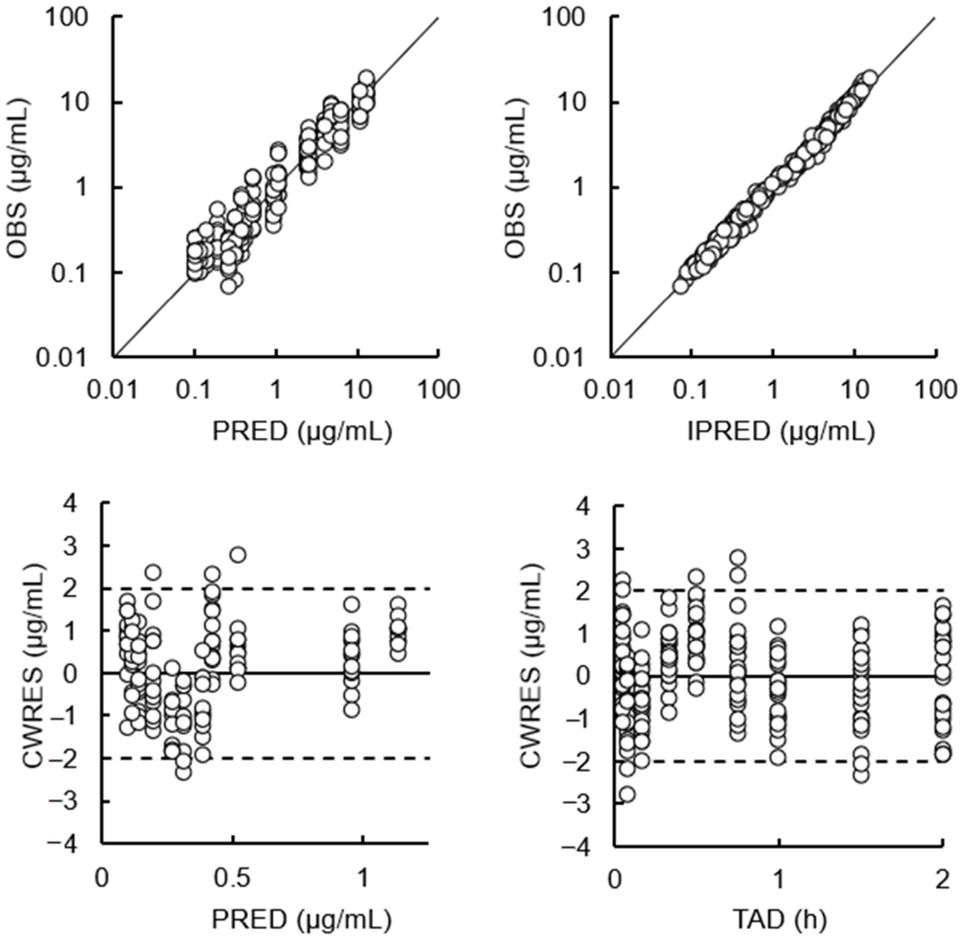

Figure S1. Final pharmacokinetics model diagnostic plots of the observed versus predicted concentrations and the conditional residuals versus predicted concentrations.
